# Translation and Validation of the Motivation to Change Lifestyle and Health Behaviours for Dementia Risk Reduction (MCLHB-DRR) Questionnaire among the General Israeli Population

**DOI:** 10.3390/ijerph20032622

**Published:** 2023-02-01

**Authors:** Anastasia V. Shvedko, Yuval Versolker, Offer E. Edelstein

**Affiliations:** The Spitzer Department of Social Work, Ben-Gurion University of the Negev, Beer-Sheva 841050, Israel

**Keywords:** dementia, motivation, lifestyle change, health behaviours, risk reduction, validation study

## Abstract

Objective: The need to promote awareness of dementia prevention is broadly emphasized in Israel. Currently, there is no valid version of a Hebrew questionnaire to assess attitudes and beliefs related to dementia prevention. This study aimed to translate and validate the MCLHB-DRR questionnaire among the general Israeli population. Methods: A total sample of 328 participants between the ages of 50–83 years (mean = 58.7, SD = 6.9) were included in this study. Participants completed the online translated MCLHB-DRR questionnaire. Exploratory factor analyses (EFA) and confirmatory factor analyses (CFA) were conducted to assess the questionnaire’s validity. Internal consistency was assessed using Cronbach’s alpha. Results: The EFA analysis revealed a seven-factor model with 27 items. One item related to perceived barriers and two items related to perceived severity were deleted. The CFA analysis confirmed a good model fit with the deleted items (χ^2^/df = 2.146, CFI = 0.930, TLI = 0.916, RMSEA = 0.049). Cronbach’s alpha values ranged from 0.61 to 0.92. Conclusions: The Hebrew MCLHB-DRR questionnaire is a valid and reliable measurement tool for assessing attitudes and beliefs related to health behaviours and lifestyle changes for dementia risk reduction in Israeli adults over the age of 50.

## 1. Background

*Dementia* is a major public health concern affecting around 55 million people worldwide [1]. This number is expected to rise to 152 million by 2050 [2]. In Israel, dementia was the third leading cause of death among the elderly in 2019 [3], and its prevalence is projected to rise to 290,000 cases in 2030 [4]. In Israel, due to a high (97%) prevalence of people aged 65 and over living in the community, the impact of dementia on health and the development of health services for dementia prevention is broadly emphasized [5]. Dementia, recognised as a syndrome, primarily affects older adults, but is not only limited to this population [1]. It is highly prevalent in women [2], and higher in Black and Asian ethnic minority groups [6,7]. Dementia is considered to be one of the risk factors for morbidity, the seventh cause of all-cause mortality, and the fourth cause of death among people 70 years of age and older globally [8].

Given the growth of the world’s ageing population and the economic, health, and societal burden of dementia, early prevention of risk of dementia is highly prevalent in health research [2]. According to specialists, the assessment of dementia should incorporate multi-domain measures combining non-modifiable (age, genetics) and modifiable factors (socio-demographic, health, and lifestyle) [9]. Modifiable risk factors accounted for up to 48% of the risk of dementia onset [10], resulting in considerable research efforts on health and lifestyle changes that can reduce this risk. These risk factors, include alcohol consumption, obesity, hearing loss, traumatic brain injury, and hypertension [11]. Other modifiable risk factors include social isolation, diabetes, and physical inactivity [10]. On the contrary, beneficial lifestyle changes can reduce the risk of dementia, including regular exercise, an optimal diet, stimulating cognitive activity, and moderate alcohol consumption [10].

The original version of the Motivation to Change Lifestyle and Health Behaviours for Dementia Risk Reduction (MCLHB-DRR) questionnaire was developed by Kim et al. [12] in Australia. This questionnaire includes 27 items across seven subscales reflecting the seven concepts of the health belief model (HBM) [13], and measures attitudes and beliefs related to health behaviours and lifestyle changes for dementia risk reduction. It was shown to be reliable and valid for adults aged 50+ years among the Australian population (the Cronbach’s alpha ranges from 0.608 to 0.864) [12], for adults between 30 and 80 years old among the Dutch population (the Cronbach’s alpha ranges from 0.69 to 0.93) [14], and for people aged 40+ years among the Turkish population (the Cronbach’s alpha ranges from 0.682 to 0.847) [15].

Understanding the importance of prevention and reduction of dementia in at-risk populations has led to a variety of multi-centre lifestyle interventions. The scale’s effectiveness in addressing dementia was shown in the Finnish Geriatric Intervention Study (FINGER) [16], the Healthy Ageing Through Internet Counselling in the Elderly (HATICE) trial [17], the Multidomain Alzheimer Preventive Trial (MAPT) [18], and the Prevention of Dementia by Intensive Vascular Care (PreDIVA) trial [19]. Findings from these studies showed that multidomain interventions that include a combination of healthy lifestyle factors, such as physical activity, cognitive training, a healthy diet or nutritional advice, social stimulation, or internet counselling, may contribute to cognitive performance in at-risk older adults [16,17,19]. In addition, future studies should investigate the potential associations between vascular factors (e.g., hypertension, obesity, hypercholesterolemia, etc.), cognitive perceptions, and participation in multidomain interventions [16,17,19]. Social cognitive theories and models suggest that health behaviour is a stage-based, complex cognitive process that involves attitudes and beliefs about behaviour change, such as an individual’s current stage of change, reinforcement management, and perceived benefits and barriers [5]. To the best of our knowledge, there is no tool that measures attitudes and beliefs related to health behaviours and lifestyle changes for dementia risk reduction in the Israeli population. Nevertheless the MCLHB-DRR [12] questionnaire can contribute to the development of future health interventions and community programmes for dementia prevention in society. This study sought to translate and validate the MCLHB-DRR questionnaire among the general Israeli population, and to explore the factors affecting attitudes and health beliefs concerning behavioural and lifestyle changes for the risk of dementia reduction in middle-aged and older adults.

## 2. Materials and Methods

### 2.1. Participants and Data Collection

This was a descriptive and cross-sectional study conducted in Israel from October 2021 to August 2022. Convenience sampling was used. Inclusion criteria for study participation were: (a) to be community-dwelling individuals; (b) aged 50 years and above without dementia or cognitive impairment; and (c) proficient in the Hebrew language. Participants with a diagnosis of dementia or psychiatric disorders and cognitive impairment were excluded from the study. Using social media forums and groups dedicated to the older adult population, we provided the potential participants with an invitation and link to participate in the anonymous online survey voluntarily. We collected data using Google Docs software (version 249.0, Google LLC, Mountian View, CA, USA). The study aims were explained in the invitation and were repeated on the first page of the online survey. No personal details such as names, identification numbers, phone numbers, or other disclosing information were collected. In order to secure the participants’ privacy, the survey data were coded anonymously to a password-protected file. The study protocol was approved by the Research Ethics Committee of the Social Work department, Ben-Gurion University of the Negev, Israel. Written informed consent was obtained from all participants prior to participation.

#### Measures

Participants completed a socio-demographic form and the translated 27-item MCLHB-DRR questionnaire.

### 2.2. The Socio-Demographic Form

Participants provided socio-demographic information about their age, gender, marital status, level of education, number of children, religiosity, area of residence, level of income, and a history of dementia such as acquaintance with an individual diagnosed with dementia, having a family member diagnosed with dementia, or providing care for an individual with dementia.

### 2.3. The Motivation to Change Lifestyle and Health Behaviours for Dementia Risk Reduction (MCLHB-DRR) Questionnaire

The Hebrew version of the original 27-item MCLHB-DRR questionnaire was developed in this study. The health belief model (HBM) served as a theoretical framework underpinning the study, applied to understanding attitudes and motivation to change behavioural and lifestyle factors to reduce the risk of dementia. The HBM posits that the threat of developing a health condition can be a motivating stimuli for making prudent, cost-effective choices through perceived susceptibility and perceived severity, the potential health benefits of which outweigh perceived barriers to health-promoting behaviours [20]. The MCLHB-DRR questionnaire includes 27 items reflecting seven subscales of the health belief model to health and lifestyle behaviour change for risk of dementia reduction: perceived susceptibility (4 items), perceived severity (5 items), perceived benefits (4 items), perceived barriers (4 items), cues to action (4 items), general health motivation (4 items), and self-efficacy (2 items). All items were rated on a 5-point Likert scale ranging from 1 (strongly disagree) to 5 (strongly agree), with higher scores representing stronger beliefs for behaviour and lifestyle change for risk of dementia reduction for all subscales apart from perceived barriers. The MCLHB-DRR questionnaire has demonstrated good internal consistency with Cronbach’s alpha from 0.608 to 0.864 [12,15,21], moderate test-retest reliability for all subscales (Cronbach’s alpha from 0.552 to 0.776), and high reliability and internal consistency irrespective of age and gender in previous research [12].

### 2.4. Scale Translation

To ensure language equivalence, the development of the Hebrew version of the MCLHB-DRR questionnaire followed a multi-stage process [22]. First, three team members, proficient in both Hebrew and English, independently translated the MCLHB-DRR questionnaire into Hebrew. Second, professional translators combined all three versions into one Hebrew version, and any discrepancies were resolved during a discussion, taking into account the original questionnaire. Third, the translation of the Hebrew version back to English was carried out by two uninformed professional translators (i.e., not familiar with the researched concepts) to check for any discrepancies from the original tool. Then, all produced versions were reviewed by informed translators (i.e., familiar with the researched concepts), who compared the translations’ accuracy. Any semantic differences found in the translations were resolved by a third-party discussion. After creating the pre-final version of the questionnaire, it underwent preliminary testing for clarity of expression and content validity with a total of 30 community-dwelling older adults without dementia or cognitive decline, who were recruited by the research team network. These participants were not included in the statistical analysis. Any issues were resolved by consensus among all of the contributors. Finally, two back translations were combined into a final Hebrew version of the MCLHB-DRR questionnaire.

#### Statistical Analysis

Baseline descriptive statistics were calculated for socio-demographic characteristics and the MCLHB-DRR questionnaire. To determine the structural validity, we conducted an exploratory factor analysis (EFA) using principal component factor analysis. The oblique rotation method was chosen as a primary method. However, if data was not normally distributed or the correlations between all factors were below 0.32 [23], the varimax rotation method was used instead. Items were deleted if inter-correlations between any items were less than 0.20. A careful examination of any item with correlations of >0.7 was considered [24]. The number of factors to be retained was observed by positive eigenvalues greater than 1.00 [25] and visual observation of a scree-plot. Additionally, the parallel analysis empirically estimated the final number of factors to retain from the principal component factor analysis [26]. Any items with a factor loading below 0.30 were deleted immediately. A rotated factor loading of a minimum of 0.512 within each factor was considered statistically significant [27]. Significant cross-loading was indicated by a difference of a minimum 0.2 between item loadings [28]. If the difference was >0.2, the highest loading was interpreted as a factor and the item was retained [29]. Values with a low rotated factor loading (<0.512) and a significant cross-loading were considered for deletion. Internal consistency of the subscales (Cronbach’s alpha and item-total correlations) was calculated. A Cronbach’s alpha of ≥0.7 indicated a good internal consistency [24]. Items with an item-total correlation below 0.30 were considered for deletion [30].

Construct validity of the MCLHB-DRR questionnaire was assessed using confirmatory factor analysis (CFA). Multiple fit indices evaluated the goodness-of-fit model: (1) χ^2^ and its degree of freedom (df), (2) the root mean square error of approximation (RMSEA), (3) Tucker–Lewis Index (TLI), and (4) the comparative fix index (CFI). An acceptable model fit was defined as χ^2^/df < 3 [31], RMSEA < 0.05 (excellent) to <0.09 (moderate) [31], TLI > 0.90 (moderate) to >0.95 (excellent), and CFI > 0.90 (moderate) to >0.95 (excellent) [32].

Descriptive statistics and EFA were analysed using SPSS version 21.0 for Windows (SPSS Inc., Chicago, IL, USA). CFA was performed using Amos version 20.0 to check the scale’s factor structure. The alpha level was set at *p* < 0.05. Participants with any missing responses on the questionnaire were excluded from the data analysis.

## 3. Results

### 3.1. Participants and Recruitment

A total sample of 328 participants (the mean age = 58.7, SD = 6.9, range 50–83 years), 66.2% female, from different regions of Israel was recruited for this study. Table 1 shows the participants’ socio-demographic characteristics.

The majority of the participants were married (81.1%), had on average three children (SD = 1.4), and had an average of 16 years’ education (SD = 3.0). The majority of the participants were secular (64.9%). About half of the participants lived in the central part of Israel (50.3%). About 51.8% of the participants reported having an above-average income; a larger percentage of males (73%) reported having a combined (above average and way above average) income level. Over half of the participants reported having an acquaintance with an individual diagnosed with dementia (57.3%), relatives/friends with dementia (51.5%), and 21.0% reported being a caregiver of relatives/friends diagnosed with dementia. A combined level of above average and way above average income was higher among males (73%) compared to females (64%); however, a larger proportion of females (34.6%) than males (23.4%) said they had an average income. Differences in the level of income between men and women were not significant (χ^2^ = 5.18, *p* = 0.269). The number of females was almost double that of males for the 50- to 60-year age category (69.2% female, 30.8% male), and for the 60- to 70-year age category (66.3% female, 33.7% male). In the 70-year-old and over age group, the number of females was 46.9% and 53.1% were male. Significant differences between men and women were found for age (t(326) = 2.38, *p* = 0.018), acquaintance of individual diagnosed with dementia (χ^2^ = 8.87, *p* = 0.003), and caring for relatives/friends diagnosed with dementia (χ^2^ = 7.17, *p* = 0.007). No significant differences were observed between men and women for other demographic characteristics (*p* < 0.05).

### 3.2. Exploratory Factor Analysis for the MCLHB-DRR Questionnaire

First, the EFA was performed using principal component analysis and varimax rotation as correlations between all factors were below 0.32. The data was adequate for data analysis as indicated by the significant Bartlett’s test for sphericity (χ^2^(351) = 4053.124, *p* < 0.005); the Kaiser–Meyer–Okin (KMO) coefficient of 0.847 exceeded 0.5; and the anti-image matrix of covariances and correlations > 0.5, indicating the adequate sample size [24,33]. Inter-item correlation was 0.87 (*p* < 0.001) between Items 1 and 2, 0.80 (*p* < 0.001) between Items 1 and 3, and 0.91 (*p* < 0.001) between Items 2 and 3. Although these items showed high inter-item correlations, they were retained as they were loaded on their intended factors and measured something else (r < 0.90). The principal component analysis with the varimax rotation showed convergence for seven factors with eigenvalues greater than one. The total explained variance of the seven factors was 67.51%. The cumulative percentages of explained variance were as follows: 25.26% by the first factor, 13.70% by the second factor, 7.98% by the third factor, 6.86% by the fourth factor, 5.63% by the fifth factor, 4.27% by the sixth factor, and 3.82% by the seventh factor. The visual observation of a scree plot also suggested a seven-factor model. Item 15 had a significant cross-loading as indicated by the calculated difference between the highest and second-highest loading for an item < 0.2; therefore, it was deleted [29]. All items were loaded on their intended subscales (Table 2). Item 6 had a low factor loading (<0.3) and was deleted. Item 8 had a low factor loading (<0.512) and was considered for deletion. The inter-scale correlations ranged from 0.10 to 0.48.

### 3.3. Analysis of the MCLHB-DRR Questionnaire’s Psychometric Characteristics

The mean (SD; range) scores of the different MCLHB-DRR subscales were as follows (Table 3): perceived susceptibility—9.1 (3.9; 4 to 20); perceived severity—14.4 (4.3; 4 to 25); benefits—14.5 (3.8; 4 to 20); barriers—7.9 (3.3; 4 to 18); cues to action—10.0 (4.4; 4 to 20); general health motivation—14.4 (3.1; 4 to 20); and self-efficacy—4.0 (2.1; 2 to 10). No significant differences were observed between men and women for all subscales (*p* < 0.001). The item response score ranged from 1.6 (0.8; item 15) to 4.2 (1.0; item 22).

### 3.4. Internal Consistency

An item-total correlation analysis showed a positive correlation of items with the total MCLHB-DRR questionnaire; however, items 8 (r = 0.23), 9 (r = 0.26), and 22 (r = 0.17) had correlations below 0.30. Item 8 was deleted because of a low factor loading (<0.512) and low correlations. Cronbach’s alpha values for subscales are presented in Table 3. Cronbach’s alpha values for perceived susceptibility was α = 0.920; for perceived severity—α = 0.610; for perceived benefits—α = 0.800; for perceived barriers—α = 0.766; for cues to action—α = 0.869; for general health motivation—α = 0.700; and for self-efficacy—α = 0.670.

### 3.5. Confirmatory Factor Analysis (CFA)

**Figure 1:**
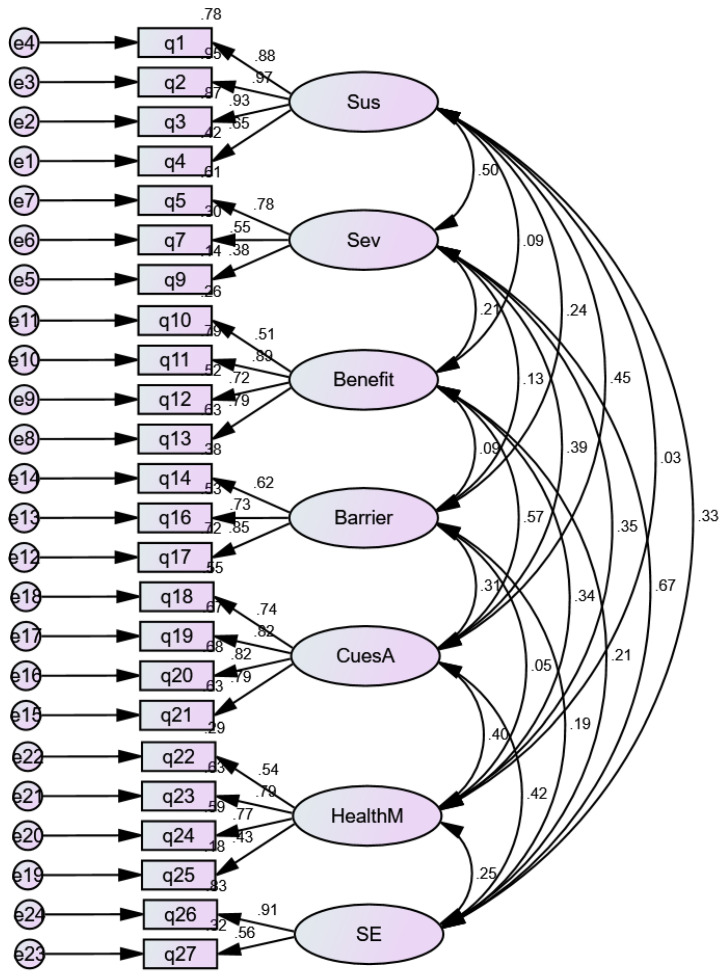
CFA model with 24 items. Sus (perceived susceptibility), Sev (perceived severity), Benefit (perceived benefits), Barrier (perceived barriers), CuesA (cues to action), HealthM (general health motivation), SE (self-efficacy).

We assessed the model’s fit using CFA with the maximum likelihood method (Table 4). The initial analysis of a seven-factor model with all 27 items (Model 1) did not demonstrate a good fit (χ^2^/df = 2.709, CFI = 0.874, TLI = 0.843, RMSEA = 0.072). Consequent analysis of a seven-factor model without items 6, 8, and 15 (Model 2) demonstrated a good fit (χ^2^/df = 2.146, CFI = 0.930, TLI = 0.916, RMSEA = 0.049), suggesting that Model 2 displays a better fit to the data than Model 1 (Figure 1, CFA model with 24 items). The factor loadings for Model 2 ranged from 0.375 to 0.975 (Table 5).

## 4. Discussion

The aim of this study was to translate and validate the MCLHB-DRR questionnaire among the general Israeli population. EFA showed that the seven-factor model, reflecting seven subscales of the MCLHB-DRR, had one cross-loaded item that was deleted (Item 15). Almost all items were loaded on their intended subscales with factor loadings of above 0.3. Two items (Items 6 and 8) were deleted due to low factor loadings and low correlations. The conducted CFA showed that a 24-item model (without Items 6, 8 and 15) was a better fit for the data than the 27-item model (χ^2^/df = 2.146, CFI = 0.930, TLI = 0.916, RMSEA = 0.049). The value of χ^2^/df = 2.146 was higher than that among the Dutch general population (χ^2^/df = 2.130), the value of CFI = 0.930 was higher compared to that among the Australian population (CFI = 0.920), the RMSEA value of 0.049 was slightly above that indicated by (RMSEA = 0.047). The internal consistency reliability for subscales of the MCLHB-DRR questionnaire ranged from Cronbach’s alpha values of 0.610 to 0.920 (moderate to high). Out of the total three excluded items, two items were from the perceived severity subscale. These emotionally driven items are related to fear, which, according to previous research, tends to be higher among females, people with higher education, and poor self-rated health [34]. Consequently, in this study lower Cronbach’s alpha values can be attributed to population differences regarding perceptions of dementia at the personal level and discrepancies in personal knowledge regarding dementia between people of different socioeconomic or gender groups. The results of this study suggest that the Hebrew version of the Motivation to Change Lifestyle and Health Behaviours for Dementia Risk Reduction (MCLHB-DRR) questionnaire is a valid and reliable tool for the assessment of attitudes and beliefs related to lifestyle and health behaviour changes for dementia risk reduction in people aged 50 years and above. These findings are not surprising, considering that dementia is associated with high fear levels and recent statistics show an increase in dementia diagnosis among individuals younger than 60 [34]. Fear is a prominent predictor of lifestyle and habit changes [34]. However, the current study had an unequal spread between male and female participants on items that were designed to measure individuals’ motivation to change lifestyle and health behaviours in order to reduce dementia, which may impact factor loadings.

Another point to consider which may have affected the results of the current study is the correlation between gender and socioeconomic status concerning dementia. According to a nationwide, population-based study, higher rates of dementia were observed among females and in people with a higher socioeconomic status. In the current study, more than half of the participants reported a high level of income and a mean number of 16.0 ± 3.0 years of education. Item 15—“My financial situation doesn’t allow me to change my lifestyle and behaviour”—had a significant cross-loading. It might be assumed that the larger proportion of females in this study and their reported, mainly average (34.6%), income level versus that of the males (23.4%) is related to fears of socioeconomic instability to change the motivation for lifestyle and health beliefs to reduce dementia, despite the fact that we did not find statistically significant differences between genders concerning income level. Findings of the current study highlight similarities and differences between our sample and those of the Australian study population [12]. Generally, the Israeli sample demonstrated lower scores than those obtained in other studies. Moreover, due to low factor loadings, the emotionally driven, fear-related Items 6 and 8 were deleted from the final version of the Israeli questionnaire. These differences can be attributed to increased awareness about dementia among the Israeli population [35]. These findings reinforce one of the major aims of the Israeli National Strategy: to disseminate information on dementia in a culturally adapted manner [35].

## 5. Limitations

The current study has several limitations: First, the study is based on a convenience sample and therefore may not represent or be generalised to the entire Israeli population. Future studies should address this limitation and use representative sampling to decrease the probability of a sampling error and to generalise the study findings to the population at large. Second, we translated a questionnaire from a previously validated English version of the MCLHB-DRR. This raises the problem of ethnocentricity and rejects the assumption of a primary language (i.e., translating a questionnaire word-by-word literally from the original version versus creating the questionnaire from a primary language maintaining the main meaning of the items) [36]. Third, the response rate cannot be calculated in this study, as we used social media for data collection, which may limit the generalisability of interpreting the results. This should be addressed in future studies by using different sampling techniques. Fourth, we cannot conclude what lifestyle and behaviour change strategy participants were informed about related to reducing the risk of dementia, nor how the absence of this knowledge may have influenced their answers. Future research is recommended to provide health professionals with a deeper understanding of prior dementia-related knowledge and risk/motivation perceptions. Fifth, although the study had an adequate sample of 328 participants, it is somewhat modest. However, based on the assumptions of a factor analysis, sample size can be at least 300 participants [33]. In addition, as a “rule of thumb”, a minimum of 10–15 participants should be adequate for each item of a factor analysis, which ranges from 270 to 405 participants in our study based on a total of 27 items of the MCLHB-DRR questionnaire to satisfy the participant assumption of the factor analysis [33]. Moreover, an equal spread of female and male participants is desirable in future studies. Our study is characterised by a relatively high proportion of non-secular participants (35.1%). According to the literature, religious individuals may lead a lifestyle that includes a strict diet, a sedentary lifestyle, and the under-usage of medical services, all of which may increase their risk of dementia compared to non-religious individuals [37]. Therefore, study findings may be affected by the responses of non-religious participants, who may have a better knowledge of health behaviours to decrease the risk of dementia development. Nevertheless, future studies should explore complex associations between religion and dementia awareness and consider informing participants about dementia prevention health behaviours before answering the MCLHB-DRR questionnaire. Another limitation of the current study is a relatively low percentage (3.3%) of widows/widowers compared to married participants. According to research, social interaction level and day-to-day cognitive stimulation are lower among individuals who have experienced the loss of meaningful others [38]. Widowers may also be less aware of dementia due to limited social support and feelings of loneliness and social isolation [38], as well as limited education and access to healthcare [39]. This may have some impact on the study findings. Despite the above limitations, the current study is the first to analyse the psychometric properties of a Hebrew translation of the MCLHB-DRR questionnaire, which measures attitudes and beliefs for health behaviours and lifestyle changes for dementia risk reduction among the general Israeli population. The translation of the questionnaire was carried out in accordance with the multi-stage process, and the Hebrew version can be used in future intervention studies to prevent dementia in the Israeli population.

## 6. Implications and Recommendations for Future Research

The exploratory and confirmatory analysis performed in the current study may be used in feasibility interventions to test different efficacy outcomes related to motivation for health and lifestyle behavioural changes to reduce dementia in older adults before proceeding to large-scale studies. Future research should attempt to address issues of participant knowledge about some concepts related to the research topic in order to bring higher awareness to the answered questions.

The validation of the Hebrew version of the MCLHB-DDR contributes to developing future intervention and cross-sectional studies that assess health beliefs and behavioural changes to reduce dementia in community-dwelling older adults. The reliability analysis of the Hebrew MCLHB-DRR questionnaire provides valuable information for developing future health research and promotes health specialists’ understanding regarding factors associated with dementia-related motivation for health behaviour and lifestyle change. Future research should consider the applicability of research to different ethnic groups within the general population.

## 7. Conclusions

The validation of the Hebrew MCLHB-DRR questionnaire in community-dwelling older adults demonstrated that the 24-item form is a reliable and valid method with which to assess attitudes and beliefs for health behaviour and lifestyle change for dementia risk reduction in the Israeli population aged 50 and above. The questionnaire can facilitate future intervention studies in health research designed to prevent dementia in community-dwelling adults, and inform about attitudes and beliefs regarding health and lifestyle changes required to reduce dementia.

## Figures and Tables

**Table 1:** Characteristics of the sample.

Characteristics	All Participants (*N* = 328) ^a^
Age (years), mean (SD)	58.7 (6.9)
Number of children, mean (SD)	3.0 (1.4)
Number of years in education, mean (SD)	16.0 (3.0)
Gender, *n* (%)	
Female	217 (66.2)
Male	111 (33.8)
Marital status, *n* (%)	
Married	266 (81.1)
Divorced	42 (12.8)
Single	7 (2.1)
Widow/Widower	10 (3.3)
Other	3 (0.9)
Income level, *n* (%)	
Way above average	42 (12.8)
Above average	170 (51.8)
Average	101 (30.8)
Below average	12 (3.7)
Way below average	3 (0.9)
Religiosity, *n* (%)	
Secular	213 (64.9)
Non-secular	115 (35.1)
Area of residency, *n* (%)	
the South	111 (33.8)
the Centre	165 (50.3)
the North	46 (14.0)
Elsewhere	6 (1.8)
Acquaintance with an individual diagnosed with dementia, *n* (%)	188 (57.3)
Having relatives/friends with dementia, *n* (%)	169 (51.5)
Providing care for relatives/friends diagnosed with dementia, *n* (%)	69 (21.0)

^a^ The number of participants (percentages) are as shown, unless otherwise stated.

**Table 2:** Exploratory factor analysis for the MCLHB-DRR questionnaire (*N* = 328, Principal components with Varimax rotation).

		Factor 1	Factor 2	Factor 3	Factor 4	Factor 5	Factor 6	Factor 7
Q1	My chances of developing dementia are great	**0.887**	−0.008	0.154	0.051	−0.021	0.115	0.029
Q2	I feel that my chances of developing dementia in the future are high	**0.913**	−0.007	0.173	0.129	−0.028	0.104	0.005
Q3	There is a strong possibility that I will develop dementia	**0.895**	0.013	0.176	0.088	−0.078	0.123	0.084
Q4	Within the next 10 years I will develop dementia	**0.723**	0.074	0.157	0.023	−0.015	0.045	0.321
Q5	The thought of dementia scares me	0.363	0.107	−0.089	−0.017	0.356	**0.588**	0.192
Q6	When I think about dementia my heart beats faster	−0.045	−0.005	0.310	−0.042	0.079	0.260 *	−0.002
Q7	My feelings about myself would change if I develop dementia	0.214	0.037	0.104	0.054	0.017	**0.729**	0.077
Q8	When I think about dementia I feel nauseous	−0.012	0.033	0.181	−0.215	0.116	**0.405**	−0.023
Q9	It would be more serious for me to develop dementia than if I developed other diseases	−0.010	0.054	0.130	0.037	−0.071	**0.682**	0.129
Q10	Information and advice from experts may give me something that I never thought of, and may reduce my chance of developing dementia	0.103	**0.538**	0.041	0.056	0.081	0.189	0.324
Q11	Changing my lifestyle and health habits can help me reduce my chance of developing dementia	0.066	**0.862**	0.103	−0.013	0.083	0.058	0.057
Q12	I have a lot to gain by changing my lifestyle and health behaviour	0.069	**0.711**	0.039	0.177	0.223	0.158	−0.123
Q13	Adapting to a healthier lifestyle and behaviour would prevent dementia for me	−0.023	**0.822**	0.222	0.079	0.051	−0.130	0.108
Q14	I am too busy to change my lifestyle and health habits	0.151	0.026	0.049	**0.758**	−0.087	0.131	−0.033
Q15	My financial situation does not allow me to change my lifestyle and health behaviour	0.239	−0.009	0.006	**0.438**	−0.003	−0.246	**0.559**
Q16	Family responsibilities make it hard for me to change my lifestyle and behaviour	0.022	−0.021	0.152	**0.805**	0.022	−0.078	0.236
Q17	Changing lifestyle and behaviour interferes with my schedule	0.040	−0.002	0.139	**0.852**	0.034	0.065	0.015
Q18	Being forgetful makes me think I have to change my lifestyle and behaviour	0.170	0.166	**0.733**	0.226	0.072	0.135	0.114
Q19	Having risk factor(s) for dementia makes me think I have to change my lifestyle and behaviour	0.378	0.214	**0.702**	0.070	0.162	0.098	0.082
Q20	Learning more about dementia from the media makes me think I have to change my lifestyle and behaviour	0.176	0.345	**0.723**	0.112	0.157	0.077	0.192
Q21	Knowing family member(s) with dementia makes me think I have to change my lifestyle and behaviour	0.224	0.328	**0.715**	0.092	0.200	0.059	−0.009
Q22	Nothing is as important to me as good health	−0.230	0.098	0.217	−0.118	**0.598**	−0.090	0.177
Q23	I often think about my health	−0.039	0.184	0.077	−0.005	**0.832**	0.005	0.100
Q24	I think I have to pay attention to my own health	0.000	0.124	0.120	−0.018	**0.830**	0.061	−0.095
Q25	I am concerned about my health	0.371	0.170	0.108	0.228	**0.452**	0.160	−0.005
Q26	I am certain that I can change my lifestyle and behaviour so I can reduce the risk of developing dementia	0.217	0.066	0.140	0.009	0.205	0.366	**0.632**
Q27	I am able to make differences that will change the risk of developing dementia	0.036	0.080	0.154	0.079	−0.002	0.280	**0.729**

Factor loadings of the MCLHB-DRR questionnaire above 0.3 are shown in bold. * Item 6 had a below 0.3 factor loading.

**Table 3:** Means and internal consistency of the subscales (*N* = 328) ^a^.

Subscale	Hebrew MCLHB-DRR Questionnaire			
	No. of Items	Range of Scores	Mean (SD)	Cronbach’s Alpha
Perceived susceptibility	4	4–20	9.1 (3.9)	0.920
Perceived severity	3	3–15	10.5 (3.0)	0.610
Perceived benefits	4	4–20	14.8 (3.8)	0.800
Perceived barriers	3	3–15	6.3 (2.9)	0.766
Cues to action	4	4–20	10.0 (4.4)	0.869
General health motivation	4	4–20	14.4 (3.1)	0.700
Self-efficacy	2	2–10	4.0 (2.1)	0.670

^a^ Items 6,8 and 15 were deleted. SD—standard deviation.

**Table 4:** Goodness of fit indices for Hebrew MCLHB-DRR model.

	χ^2^/df	TLI	CFI	RMSEA
Hebrew MCLHB-DRR Model 1	2.709	0.843	0.874	0.072
Hebrew MCLHB-DRR Model 2	2.146	0.916	0.930	0.049

Model 1 represents a seven-factor model with all 27 items. Model 2 represents a seven-factor model with 24 items (without items 6, 8 and 15). Abbreviations: RMSEA, root mean squared error of approximation; CFI, comparative fit index; TLI, Tucker–Lewis Index; MCLHB-DRR, motivation to change lifestyle and health behaviours for dementia risk reduction.

**Table 5:** Confirmatory Factor Analysis report.

Subscales	Item	Factor Loading
Perceived susceptibility	Q1	0.883 *
	Q2	0.975 *
	Q3	0.934 *
	Q4	0.650 *
Perceived severity	Q5	0.779 *
	Q7	0.550 *
	Q9	0.375 *
Perceived benefits	Q10	0.511 *
	Q11	0.890 *
	Q12	0.720 *
	Q13	0.791 *
Perceived barriers	Q14	0.617 *
	Q16	0.729 *
	Q17	0.850 *
Cues to action	Q18	0.739 *
	Q19	0.817 *
	Q20	0.822 *
	Q21	0.794 *
General health motivation	Q22	0.543 *
	Q23	0.793 *
	Q24	0.765 *
	Q25	0.425 *
Self-efficacy	Q26	0.914 *
	Q27	0.564 *

Results are shown for Model 2, which represents a seven-factor model with 24 items (without items 6, 8 and 15). * *p* < 0.001.

## Data Availability

The translated Hebrew MCLHB-DRR questionnaire is available from the corresponding author upon request.

## Abbreviations

MCLHB-DRR	motivation to change lifestyle and health behaviours for dementia risk reduction
EFA	exploratory factor analysis
CFA	confirmatory factor analysis
TLI	Tucker–Lewis index
RMSEA	root mean square error of approximation
HBM	health belief model
KMO	Kaiser–Meyer–Okin
SD	standard deviation

## References

[1] WHO Dementia 2021. https://www.who.int/news-room/fact-sheets/detail/dementia.

[2] Nichols E., Steinmetz J.D., Vollset S.E., Fukutaki K., Chalek J., Abd-Allah F., Abdoli A., Abualhasan A., Abu-Gharbieh E., Akram T.T. (2022). Estimation of the global prevalence of dementia in 2019 and forecasted prevalence in 2050: An analysis for the Global Burden of Disease Study 2019. Lancet Public Health.

[3] (2019). Causes of Death in Israel-Interactive Display System. https://statistics.health.gov.il/views/DeathCauses/Leadingcausesofdeathbyage?%3Aembed=y&Language%20Desc=English.

[4] Bentur N., Sternberg S.A. (2019). Dementia care in Israel: Top down and bottom up processes. Isr. J. Health Policy Res..

[5] Glanz K. (2016). Behavioural & Social Sciences Research. https://obssr.od.nih.gov/wp-content/uploads/2016/05/Social-and-Behavioral-Theories.pdf.

[6] Shiekh S.I., Cadogan S.L., Lin L.-Y., Mathur R., Smeeth L., Warren-Gash C. (2021). Ethnic Differences in Dementia Risk: A Systematic Review and Meta-Analysis. J. Alzheimer’s Dis..

[7] Co M., Couch E., Gao Q., Martinez A., Das-Munshi J., Prina M. (2021). Differences in survival and mortality in minority ethnic groups with dementia: A systematic review and meta-analysis. Int. J. Geriatr. Psychiatry.

[8] Collaborators G., Nichols E., Abd-Allah F., Abdoli A., Abosetugn A.E., Abrha W.A., Abualhasan A., Abu-Gharbieh E., Akinyemi R.O., Alahdab F. (2021). Global mortality from dementia: Application of a new method and results from the Global Burden of Disease Study 2019. Alzheimer’s Dementia: Transl. Res. Clin. Interv..

[9] Ranson J.M., Rittman T., Hayat S., Brayne C., Jessen F., Blennow K., van Duijn C., Barkhof F., Tang E., Mummery C.J. (2021). Modifiable risk factors for dementia and dementia risk profiling. A user manual for Brain Health Services—Part 2 of 6. Alzheimer’s Res. Ther..

[10] Chong T.W., Macpherson H., A Schaumberg M., Brown B.M., Naismith S.L., Steiner G.Z. (2021). Dementia prevention: The time to act is now. Med. J. Aust..

[11] Livingston G., Sommerlad A., Orgeta V., Costafreda S.G., Huntley J., Ames D., Ballard C., Banerjee S., Burns A., Cohen-Mansfield J. (2017). Dementia prevention, intervention, and care. Lancet.

[12] Kim S., Sargent-Cox K., Cherbuin N., Anstey K.J. (2014). Development of the Motivation to Change Lifestyle and Health Behaviours for Dementia Risk Reduction Scale. Dement. Geriatr. Cogn. Disord. Extra.

[13] Guvenc G., Akyuz A., Açikel C.H. (2010). Health Belief Model Scale for Cervical Cancer and Pap Smear Test: Psychometric testing. J. Adv. Nurs..

[14] Joxhorst T., Vrijsen J., Niebuur J., Smidt N. (2020). Cross-cultural validation of the motivation to change lifestyle and health behaviours for dementia risk reduction scale in the Dutch general population. BMC Public Health.

[15] Akyol M.A., Zehirlioğlu L., Erünal M., Mert H., Hatipoğlu N., Küçükgüçlü Ö. (2020). Determining Middle-Aged and Older Adults’ Health Beliefs to Change Lifestyle and Health Behavior for Dementia Risk Reduction. Am. J. Alzheimer’s Dis. Other Dementiasr.

[16] Kivipelto M., Solomon A., Ahtiluoto S., Ngandu T., Lehtisalo J., Antikainen R., Bäckman L., Hänninen T., Jula A., Laatikainen T. (2013). The Finnish Geriatric Intervention Study to Prevent Cognitive Impairment and Disability (FINGER): Study design and progress. Alzheimer’s Dement..

[17] Richard E., Jongstra S., Soininen H., Brayne C., van Charante E.P.M., Meiller Y., van der Groep B., Beishuizen C.R.L., Mangialasche F., Barbera M. (2016). Healthy Ageing Through Internet Counselling in the Elderly: The HATICE randomised controlled trial for the prevention of cardiovascular disease and cognitive impairment. BMJ Open.

[18] Vellas B., Carrie I., Gillette-Guyonnet S., Touchon J., Dantoine T., Dartigues J.F., Cuffi M.N., Bordes S., Gasnier Y., Robert P. (2014). Mapt study: A multidomain approach for preventing Alzheimer’s disease: Design and baseline data. J. Prev. Alzheimer’s Dis..

[19] Richard E., Heuvel E.V.D., Van Charante E.P.M., Achthoven L., Vermeulen M., Bindels P.J., Van Gool W.A. (2009). Prevention of Dementia by Intensive Vascular Care (PreDIVA). Alzheimer Dis. Assoc. Disord..

[20] Glanz K., Rimer B.K., Viswanath K. (2008). Health Behavior and Health Education: Theory, Research, and Practice.

[21] Zehirlioglu L., Erunal M., Akyol M.A., Mert H., Hatipoglu N.S., Kucukguclu O. (2019). Turkish Version of the Motivation for Changing Lifestyle and Health Behavior for Reducing the Risk of Dementia Scale. J. Neurosci. Nurs..

[22] Beaton D.E., Bombardier C., Guillemin F., Ferraz M.B. (2000). Guidelines for the Process of Cross-Cultural Adaptation of Self-Report Measures. Spine.

[23] Costello A.B., Osborne J.W. (2005). Best practices in exploratory factor analysis: Four recommendations for getting the most from your analysis. Pr. Assess. Res. Eval..

[24] Field A. (2018). Discovering Statistics Using IBM SPSS statistics.

[25] Yong A.G., Pearce S. (2013). A Beginner’s Guide to Factor Analysis: Focusing on Exploratory Factor Analysis. Tutorials Quant. Methods Psychol..

[26] Patil V.H., Singh S.N., Mishra S., Donavan D.T. (2007). Parallel Analysis Engine to Aid Determining Number of Factors to Retain. 2007. [Computer Software]. https://analytics.gonzaga.edu/parallelengine/.

[27] Stevens J.P. (2012). Applied Multivariate Statistics for the Social Sciences.

[28] Byrne B.M. (2010). Testing for the factorial validity of scores from a measuring instrument (first-order CFA model). Structural Equation Modeling with AMOS: Basic Concepts, Applications, and Programming.

[29] Stamper C.L., Masterson S.S. (2002). Insider or outsider? how employee perceptions of insider status affect their work behavior. J. Organ. Behav..

[30] De Vet H.C., Terwee C.B., Mokkink L.B., Knol D.L. (2011). Measurement in Medicine: A Practical Guide.

[31] Hair J.F. (2014). Multivariate Data Analysis.

[32] Hu L.T., Bentler P.M. (1999). Cutoff criteria for fit indexes in covariance structure analysis: Conventional criteria versus new alternatives. Struct. Equ. Model. Multidiscip. J..

[33] Tabachnick B.G., Fidell L.S. (2001). Using multivariate statistics.

[34] Volicer L. (2016). Fear of Dementia. J. Am. Med. Dir. Assoc..

[35] Brodsky J.B.N., Laron M., Ben-Israel S. (2013). Addressing Alzheimer’s and other types of dementia: Israeli National Strategy. http://www.health.gov.il/PublicationsFiles/Dementia_strategy-Eng.pdf.

[36] Sperber A.D. (2004). Translation and validation of study instruments for cross-cultural research. Gastroenterology.

[37] Beeri M.S., Davidson M., Silverman J.M., Schmeidler J., Springer R.R., Noy S., Goldbourt U. (2008). Religious education and midlife observance are associated with dementia three decades later in Israeli men. J. Clin. Epidemiology.

[38] Shvedko A., Whittaker A.C., Thompson J.L., Greig C.A. (2018). Physical activity interventions for treatment of social isolation, loneliness or low social support in older adults: A systematic review and meta-analysis of randomised controlled trials. Psychol. Sport Exerc..

[39] Shin S.H., Kim G., Park S. (2018). Widowhood Status as a Risk Factor for Cognitive Decline among Older Adults. Am. J. Geriatr. Psychiatry.

